# Is Modern Education Exerting an Evil Influence upon the Eye-Sight of Your Children?

**Published:** 1878-02

**Authors:** A. W. Calhoun

**Affiliations:** Professor of Diseases of the Eye and Ear in the Atlanta Medical College. Atlanta, Georgia


ATLANTA

MEDICAL AND SURGICAL JOURNAL

VOL. XV.] FEBRUARY—1878. [No. 11

### Original Communications.

### IS MODERN EDUCATION EXERTING AN EVIL INFLUENCE UPON THE EYE-SIGHT OF YOUR CHILDREN?

BY A. W. CALHOUN, M.D.,

Professor of Diseases of the Eye and Ear in the Atlanta Medical College.

*Read before the Georgia Teachers' Association and the Atlanta Academy of Medicine.*

There have always been, and even in our own day and time there still exist, those who are in doubt as to which is the most important, the most indispensable of all our organs of sense. But especially often is the question asked, "Which is the greatest deprivation, the loss of sight or the loss of hearing?"

How almost universal is it that the blind appear bright, happy and cheerfully resigned to the great loss they have sustained, while the deaf are not unfrequently dissatisfied, unhappy and mistrustful. It has been my lot to have had much intercourse with these two classes of individuals, as well in private life as in the well-filled and well-managed institutions for their benefit throughout various portions of the world; and this remakable contrast between them is the more apparent, the more thorough and the more intimate my acquaintance grows. The doubt, indeed, is untenable, for in the eye we have that organ which brings us in the most immediate relation and connection with the outer world, with mankind, with the brute creation, with all nature; for she best expresses herself to us through her visibleness. This alone suffices for us to comprehend. Nature offers much less for the hearing, for never can we learn to conceive of objects as they really are, from the noises which may emanate from them and perceptibly strike our ear. On the contrary, imagination readily brings to mind the soft notes of the musical instrument that we *see.* We can often understand and comprehend men without hearing them, from the movements of the body and from their countenances. We can read from their lips what they speak when not a single tone is audible.

That sight is a direct aid and support to each of the other organs of sense, can be readily tested and proven by each and every one. The finest and most delicate viands never taste so well as when we *see* them. The enjoyment of the most fragrant flower is considerably heightened by the *sight* of it. A sensation, however pleasant to the touch, is infinitely increased when we *see* the cause that calls it forth.

It is in the school-room that the largest and most important portion of the child's life is spent, and while the whole energy is bent upon the proper development of the brain, it is not seldom that too little attention is given to the true maxim that "only in a healthy body can a healthy mind live." During these same years must the physical development progress, but how often can we trace back to those school days the ground-work of bodily ailments, which prove to be stubborn barriers to all future advancement.

Now, as the eye must play the role of a mediator between the subjects to be learned and the working brain, it is easy to understand that in the same proportion that work is demanded of the brain, in like degree does a tax fall upon the eye, and so it happens also that the eye, which, as the general body, is perfecting itself during these very years of school life, undergoes not unfrequently powerful changes, which we can speak of only as diseased conditions, not only not admitting of cure, but here and there leading to the destruction of the organ of sight.

After we have shown that the eye suffers such powerful and unwholesome changes during the years spent in the school-room, then it is our purpose to demonstrate that certain influences during these years *originate* diseases of the eye, and finally to enumerate the means, with the help of which we hope to be able to at least limit the frequency and degree of intensity of these diseases.

We always judge of an eye according to its length, and in eyes of different length we have different vision. In this particular we recognize three different conditions, known as conditions of refraction. These are:

1. Emmetropia or normal sightedness.
2. Myopia or near sightedness.
3. Hypermetropia or over-sightedness.

That these conditions may be clearly understood, and for a thorough comprehension of the dangers dependent upon a certain one (2d) of them, it is perhaps well that I should say a few words descriptive of them.

The hypermetropic or over-sighted eye is too short a ball, and is a congenital *defect*.

The myopic or near-sighted eye is too long a ball, and in *every* degree, from the slightest to the greatest, is not only a real *defect*, but is also an *absolute disease*. In the interior of every eye there is a circular muscle (the ciliary) known as the muscle of accommodation, by whose action we are enabled to accommodate our visions for all distances, for near, distant and all intermediate points.

In the emmetropic or normal-sighted eye, the action of this muscle produces no peculiar, no unpleasant sensations, for it acts within normal limits. But in the over-sighted or hypermetropic eye, both for seeing distinctly in the distance and very specially for seeing near objects, this little muscle must exercise excessive force, and indeed the excess of force is the greater, the more over-sighted the eye or the shorter the ball.

A muscle that is brought into constant unnatural activity gradually passes over into a convulsive, spasmodic condition, or is so much fatigued that it is no longer able to undergo work. Both of these conditions take place in the human eye. The characteristic symptoms of this condition are as follows: Such persons (adults as well as children) are able to execute the very finest work for a greater or less time, perhaps without trouble, then gradually, may be suddenly, the work becomes indistinct, obscured, and things seem to run into each other. At times it appears as if a cloud were floating immediately in front of the eye, and one is no longer able to continue the work, and as a consequence a few moments' rest follows, the eyes are rubbed, and after a pause of a short interval the work is again resumed, but mostly only for a short time, for the same symptoms return. Particularly is this the case towards evening, after an entire day's toil, or towards the end of the week, after a whole week's constant use of the eyes, while on Monday, and especially Monday morning, in consequence of resting the eye, and therefore resting the muscle of accommodation on the inside of the eye during Sunday, the vision is at its very best. In some instances, this wearied condition of the muscle appears so suddenly that the persons affected think themselves blinded, and when they seek assistance and are told that the use of proper glasses will forever remove the trouble, they are astonished as well as rejoiced.

Unfortunately, it is not seldom the case that a great wrong is done children affected with over-sightedness; parents and teachers attributing the difficulty with which they progress in their studies to a "stubborn will" or "idleness" manifesting itself in their complaints of tired eyes, inability to go on with study, headaches, and so on, when in truth it was due to the over-taxed muscle of accommodation. I have had frequent occasion to call to these facts the attention of parents and teachers whose children or pupils may have been sufferers from over-sightedness.

But it is to that condition of refraction known as myopia or near-sightedness I wish to direct attention specially, for while the over-sighted eye is an eye of defective growth without disease, the near-sighted ball is not only defective in growth, for it is too long, but is in the real sense of the term a *diseased* eye. The celebrated Donders, in his work on refraction, takes this as his motto: "I speak it without hesitation, that a near-sighted eye is a diseased eye." Let every one, then, seek diligently *not* to become near-sighted. As we have seen, the longer the eye the greater the near-sightedness, and the highest degree of it corresponds to the longest eye.

Very rarely before the fifth or sixth year of life does near-sightedness make its appearance—about which time children usually begin their attendance upon school—but from this time on, under certain unfavorable circumstances, the eye gradually elongates, reaching and remaining perhaps at a certain point of elongation, a slight or high degree, or constantly increases through all the years of school life, even to the twentieth or twenty-fifth year, and indeed in some instances continues slowly to lengthen through almost the whole life. But it is not simply the elongation of the ball that is so much to be feared, but consequent changes which have an important bearing upon the acuity of vision, upon the movements and upon the very existence of the eye.

If a hundred men with perfectly good eyes are able to recognize or read at a certain distance, say twenty feet, one and the same letter or number of letters, it is reasonable to conclude that any other person with equally good eyes could recognize and read the same letter or letters at the same distance. But if one is incapable of doing this, even with the defect of refraction (if such exists) corrected by proper glasses, then this one has less acuity of vision than the one hundred who were able to do so. This diminution of vision is a frequent and common circumstance with near-sighted individuals, and depends upon the diseased changes taking place on the interior of the eye, brought about by the abnormal lengthening of the ball.

The movements of the ball are controlled by six muscles, and over their action near-sightedness exercises a very great influence, because of the ball being so long. There exists a peculiar but certain connection between near-sightedness and the contraction of one of these muscles (the outer), causing the eye to turn outwards, constituting what in common parlance is known as *external* squint or cross eyes. A corresponding connection exists also between *over-sightedness* and *internal* squint, so that, indeed, in the majority of instances we are able to say when we see a person squinting *outwards* that he is *near-sighted*, and when *inwards* that he is *over-sighted*. There are, of course, exceptions to this rule. In every near-sighted individual the very existence of the eye is in danger, but especially so in those cases of *progressive* myopia, attaining finally a high degree. Such an eye can any moment become suddenly blinded through detachment of the retina, the lining nervous membrane on the interior of the eye, resulting always in a very material reduction of sight, and in the majority of cases in irretrievable loss of vision.

Doubtless the question has already been asked, cannot this too short eye be made longer, and this too long eye be made shorter? No, I answer; for these changes in the eye must remain forever unchanged, but we can correct the defect in the *over-sighted* eye, in so far as the unpleasant and painful symptoms from the use of such an eye are concerned, and make the vision as comfortable as in the normal-sighted person. And we can make the *near-sighted* see not only near but distant objects. It would be out of place here to go into a detailed description of the means at our command for such correction. Suffice it to say, it is done in the first instance (the over-sighted eye) by the use of convex, in the second (the near-sighted eye) by the use of concave glasses. The question naturally propounds itself, are we able to prevent this diseased lengthening of the eyeball, this near-sightedness, or after it has begun, are we in condition to at least prevent its increase? This is a question full of the most vital importance, and it is the question that has led to the investigation of the eyes of a very large number of school children, after it had once become positively known that near-sightedness developed itself *particularly*, indeed *almost exclusively*, and attained its very highest degrees *during* the years of school life. So far as I can gather, there are the records of the examinations of the eyes of about 45,000 (forty-five thousand) school children, of all ages and of all grades, representing both white and colored races, and both country and city schools. Some of these investigations would lead us to conclude that the percentage of normal-sighted, others that the percentage of over-sighted children, prevailed in the largest degree, but all, without a single exception, prove beyond a doubt that *near*-sightedness, beginning perhaps at *nothing* in the lowest classes and first years of school life, steadily increases from class to class in the school until in the highest grades or in the last years of school attendance, it has actually developed itself in as many as sixty or seventy per cent. of all the pupils. Amongst this large number we have children whose ages range all along from five to twenty years and upwards, all similarly occupied, and during a time in which the eye undergoes its most important changes—changes which will influence more or less the regulation of their entire lives.

In the schoolroom we can follow the eye from its beginning on—from its primitive condition, as it were—where as yet, it has been exposed to no injurious influences, and can follow it through a series of years to an age in which we can mark the boundary, beyond which we can say, the further development of it under certain conditions will be for good, under other conditions, for evil.

Now we have found that in the *very early* years of life, *no* eye is near-sighted, and even in the *lowest classes* at school *no* near-sightedness exists, or if at all, only in the slightest degree, and also that in the highest and last school classes, containing the oldest pupils, as much as seventy or even a larger per cent. of the scholars are near-sighted. Knowing all this, the question presses itself upon us: how is it possible for an eye in a space of time from twelve to fifteen years, to undergo such a transformation? If nature prescribes or dictates these changes in the human eye, why is it then that *all* are not near-sighted? Or do unfavorable circumstances or conditions lend their assistance in bringing about this diseased lengthening of the eyeball? Or have the sufferers inherited the disease from their parents or ancestors, and if so, why is it that these children are not near-sighted from birth?

Some very interesting observations have been made during these investigations, and prominent among them are:

1. That over-sightedness (a too short ball) is originally the normal condition of the eye, and to reach the near-sighted state (a too long ball) the eye must pass through the normal-sighted condition (a normal sized ball).

2. That with the increase in years, and with the rise in classes, the number of near-sighted children more or less rapidly increases.

3. That near-sightedness in city schools is much more frequent than in schools in the rural districts, due to the better surroundings in the country, less strain and more rest for the eye.

4. That colored school children are remarkably free from near-sightedness, the per centage being exceedingly small. In my own examinations of this class of students, I have seen very few who were near-sighted.

5. That children frequently have the trouble, whose parents or grandparents had the same, and that the acuity of vision is more frequently defective amongst the near-sighted than amongst any others.

I have before me the results of the examinations of the eyes of nearly 2,200 school children in and around Bern, the capital of Switzerland, and I take these tables because they are a fair type of all the others. These show that the majority of children begin school life with emmetropia or normal-conditioned eyes, but that the number of normal-sighted children invariably and constantly diminishes as they rise from grade to grade. They show, also, on the other hand, that myopic or near-sighted eyes scarcely exist at all, or in very small numbers, in the youngest children or in the lowest classes, but as they grow older and go higher and higher in studies requiring closer application of their eyes, just as invariably and just as constantly does near-sightedness increase, as normal-sightedness diminishes under the opposite condition of things. In similar proportion also as near-sightedness increases does over-sightedness diminish, showing beyond the shadow of a doubt that both normal-sighted and over-sighted eyes,

(with one of which almost all children are born,) under the influence of school life and other unhealthy surroundings, gradually lose their healthy condition, until finally in the last school years, and in the highest grades or classes, near-sightedness, a truly diseased condition of the eye, becomes the condition of refraction in as many as seventy per cent of those now just in the very beginning of manhood and womanhood. This is truly a sad picture and a sad commentary upon the boasted civilization of the latter half of the nineteenth century, but it is true to the very letter, and needs but a most superficial study of the subject to convince him who doubts most. But not only among school children do we find near-sightedness developing itself, but also amongst teachers, book-keepers, engravers, watch-makers, newspaper men, and indeed amongst all those who have occasion to look at fine objects for a long time, keeping the muscle of accommodation in a continued strain for a greater or less period. In all adults, however, the disease develops itself with much less frequency and with more difficulty than in children, because the tissues forming the eyeballs in the grown person have received their growth, become hardened, and are much less influenced by excessive use than those of a child.

Is it then a great misfortune to become near-sighted? Since a near-sighted eye is a diseased eye, this question bears its own answer upon its face. But what is the cause of near-sightedness, and since we cannot cure the disease—cannot make this too long ball short again—are we not able to prevent others falling into the same unfortunate condition? The two principal causes are heredity and occupation. Others may exist, but they are unimportant in comparison with these two. That over-sightedness is often hereditary is a matter of every day observation; several members of the same family not only having the defect, but oftentimes having it in exactly the same degree that existed in the eyes of their parents or grandparents. Of necessity, must hypermetropia or over-sightedness be at least congenital, but not so with myopia or near-sightedness, for it can be either hereditary or acquired. The tendency to the trouble may exist from birth, but the disease itself may never crop out unless conditions favorable to it present themselves. On the contrary also, very many children acquire near-sightedness in whom there is no natural tendency to the disease, whose parents or grand-parents were entirely free from it. It has been mentioned that in the children in country schools near-sightedness was, comparatively speaking, scarcely to be found. These pupils are the children of parents who themselves perhaps attended school but little during the early years of their lives, and had strained their eyes very little on near objects, hence the absence of an hereditary tendency to the disease in their children. The parents and grandparents of city school children have themselves, perhaps for generations back, attended city schools, and have year after year engaged in those avocations in the city which are a constant strain to the muscles (particularly the ciliary) of the eye, leading ultimately to a lengthening of the ball, a tendency to which is transmitted from generation to generation. Out of nearly five hundred (500) negro school children examined in New York by Dr. Callan, only two and a half per cent. were found near-sighed, and out of thousands of the natives of British India, examined by British surgeons, not a single one was found to be near-sighted. Amongst a large number of negroes examined by myself from time to time, only two cases of near-sightedness have as yet come under my observation. These were in grown negro men, who for eight or ten years had applied themselves unremittingly to their studies and had acquired a very extraordinary amount of intelligence, but at the cost of a defect of their eyes, which must forever leave them in a diseased state. The negroes then, are as yet but little subject to near-sightedness, because their forefathers never had occasion to use their eyes to an injurious extent, and hence, as yet also, only the tendency to good eyesight have they inherited from them.

Now, while heredity gives rise to the tendency to near-sightedness, and certain subsequent avocations cause its full development in a large number of instances, still there is no doubt whatever that the avocations of individuals during school years, as well as later, is often the sole cause of the disease, without the existence of the slightest hereditary tendency. In the schoolroom there are two kinds of influences that work injuriously upon the eyesight. Under the first are classed all those things which compel the eye to strain itself in order to see distinctly small letters or objects. Under the second, all those which cause a congestion of or rush of blood to the head and eye. To the first belong bad ventilation and improper light, too small and imperfect type, pale ink, many successive hours at the same kind of work, as in reading, writing, sewing, etc., without change or rest of the eye, all sorts of toil causing the use of the eye until late in the night and sometimes with very defective light. To the scond belong not only those things just enumerated, but also the construction and arrangement of school desks and benches, which in many schools make it next to impossible for pupils to hold their bodies in proper position for any length of time.

For demonstrating the role that bad air, bad light, etc., play in the schoolroom, I extract some pertinent remarks from a paper published by Dr. Loring, of New York. He says:

I am therefore of the opinion that bad air alone, acting as the primal cause, may set in train a series of morbid processes, which may, and often do effect, not only the working capacity and integrity of the organ, but which may lead even to its total destruction. Thus simple irritation of the mucous membrane of the eye may, and often does, pass into actual inflammation, which, increasing in violence, may proceed from part to part till the entire organ is involved, and thus the sight become impaired or totally lost.

Ought not the light to fall, not full in the face of the child, but first on the book, or work, and be reflected into the eye?

Before answering this question precisely as it now stands, I should like to premise it by making the general assertion that not only is the direction in which the light comes important, but also its quantity and quality. Reduction in illumination is, as a rule, precisely equivalent to a reduction in the size of the object. Therefore, the less the light, the nearer an object has to be brought to the eye, and the greater the strain in the act of vision.

It is impossible to fix with any scientific exactness just the size that a window should be to give sufficient light for visual purposes, since this must vary with the exposure and surroundings of the room; but it has been reckoned in Germany that for a class-room containing twenty persons, there should be at least 4,000 to 6,000 square inches of glass, which would give to each scholar from 200 to 300 square inches, or what would be represented by a pane of glass from 14 to 17 inches square. Such a room as this would be sufficiently lighted in any part. A room 20 feet square should not contain less than 70 to 80 square feet of glass, and it may be laid down as a rule that too much light cannot be obtained in a room, as all excess of glare can be guarded against by artificial shades if properly applied.

More light enters the room from the same amount of glass from the south than from the north, and a southern, southeastern or southwestern exposure is better than a northern, northeastern, or northwestern, especially for class-rooms, and this, too, simply in regard to the amount of light and independent of the purifying influences of direct sunlight.

That a north room is better for the purposes of the artist is due to other causes and does not affect the general rule.

The light should not come from directly in front, and especially is this the rule when artificial light is used. For when the light comes from directly in front of the person, the pupil of the eye becomes unduly contracted, which is equivalent to reducing the quantity of light, since less light enters the eye from the object viewed, while the eye is exposed to too much light reflected from the surrounding objects, and from the direct rays from the source of illumination.

Neither should the light come from directly behind, as the object then lies in the shadow of the body. Nor yet from the right side, because in writing the shadow of the hand falls across the page, and a moving shadow over a lighted surface not only reduces the quantity of light and leads to a stooping position, but it is also more annoying to the eye than a uniform reduction in the illumination of even a greater degree.

The best direction for the light to come is from the left-hand side, and from rather above than below the level of the head. Windows, therefore, should not be run down too near to the floor, as they often do in class-rooms and offices.

I cannot agree with the opinion often expressed, that the best direction for the light to come from is from directly above.

I cannot refrain from adding, in this connection, the conclusion founded on Dr. Cohn's elaborate investigations in regard to the near-sightedness among school children in Germany. He thus formulates it: "The narrower the street in which the school-house was built, the higher the opposite buildings, and the lower the story occupied by the class, the greater the number of near-sighted scholars."

I should, then, from these considerations, say that the angle at which the light strikes the eye is important, and that it ought not to fall in the face of the child, but first on the book or work, and be reflected into the eye.

Does the size and quality of the type cause disease of the eye?

First, in regard to size of type. The smaller an object is, the nearer it has to be brought to the eye to be perceived.

It has been accepted by occulists that type embraced by an angle equal to 5′ (five minutes) is the smallest printed matter which can be recognized by the average normal eye. According to this formula, the smallest print which a normal eye can readily recognize at a distance of one foot is about $\frac{1}{50}$ of an inch, at eighteen inches (the average distance at which the book is held by an adult,) the smallest recognizable type would be about $\frac{1}{32}$ of an inch. The normal eye should never be subjected for any length of time to a type smaller than twice this size, that is, $\frac{2}{32}$ ($\frac{1}{16}$) of an inch, and it would be better, after middle life, to employ a type even a little larger than this. The fact, however, that spectacles are now so commonly used, removes, in a great degree, by restoring to the eye its former focalizing power, the necessity of a larger type with advancing years. Young children should never hold the book nearer to the eye than ten inches, and adults never farther from the eye than eighteen inches. As soon as perfectly distinct vision at this distance cannot be obtained, and if obtained cannot be easily maintained, recourse should be had to spectacles.

The finer, then, the type, the closer the book has to be brought to the eye, and the greater the tension or demand on the focalizing power, and the muscles which are used in bringing both eyes to bear at the same time upon the object viewed. These two acts make what is called the act of accommodation of the eye, and tension of the accommodation, that is, long continued use of the eye upon objects brought close to it, is considered by all authorities one of the most, if not *the* most, fertile causes of progressive near-sightedness.

This condition may be accompanied by morbid processes, which may involve the deeper-seated membrane of the eye to such a degree as to not only affect the vision, but to destroy it. Too fine print, therefore, may be, I think, looked upon as a factor in producing eye disease, affecting not only the external, but also the internal parts of the organ.

On the other hand, too coarse print is wearisome to the eye, as it requires more exertion of the muscles governing the movements of the eye; that is, for a given amount of matter; and especially is this the case when the breadth of the page is, if anything, too great. This causes undue exertion on the part of the muscles which move the eye in a lateral direction, and is apt to lead to confusion in finding the next succeding line. It is for this reason that the narrow form of English blank verse is so little fatiguing to the eye.

The popular prejudice in regard to the double column on a page would be untenable were it not that the same economy which restricts the amount of space also reduces the size of the type and crowds the lines together. A double column page which is well printed and properly divided is certainly preferable to the same amount of matter extending in a single line across the entire page.

The distance between the lines, that is, from the bottom of the line to the top of the other, should be about $\frac{4}{32}$, or one-eighth of an inch. Nearer than this is apt to be confusing; farther, fatiguing.

QUALITY OF TYPE.—The less contrast there is between an object and its surroundings, the more difficult it is to see the object, and the closer it has to be brought to the eye. A faintly printed page has, therefore, to be brought nearer, oftentimes very much nearer, than a well-printed page of the same type. There is nothing more wearisome to an eye than an indistinct and blurred image of a familiar object, and no more striking example of this could be found than blurred or faintly printed type. This should be sharply cut, and what is technically called "heavy-faced," in contra-distinction to "light-faced" type. Of the former a common example is the English; of the latter, the American.

The ink is also a matter of importance.

English ink, like the type, is vastly superior to the American. The color and quality of the paper has also an influence upon the ease with which the act of vision is performed. For, while it is true that there should be as much contrast as possible between the type and its surroundings, care should be taken to avoid all glare or dazzling of the page. Pure white paper, such as is ordinarily used in this country, should not be employed, most of all when it has, as it often does in the cheaper papers, a metallic lustre with a bluish tinge. I have come to the conclusion that, as a rule, a very light, almost imperceptible, yellow tint is the best. This is known in the trade as "natural" tint, from the fact that it contains no dye whatever, and has been bleached only to a moderate degree. It has the color of unbleached cotton cloth. It is, however, expensive, as it can only be made from the best stock; still a very good imitation can be had in some of the second-class papers at a moderate cost. The paper should be thick enough not to be transparent, should have a close, fine texture, and be free from sponginess.

From these facts I am of the opinion that, both in respect to size and quality, imperfect type may be considered as a factor in the production of eye diseases.

Does too long and constrained attention to one object, without rest or variety, cause eye disease?

That prolonged tension of the eyes may be the primal cause of a great number of diseases of the eye is admitted by all authorities, and the more fixed the gaze and the narrower the field of view, the greater the danger. If it be true that continued tension of muscular and nervous force unduly exhausts the energy of any organ, it is doubly true of the eye. The nervous energy of the retina, sensitive and rapid as it is, is just as rapidly exhausted, and the act of reading would be unbearable after a few moments if the eye did not quickly change its position from letter to letter and from line to line. Diversity of action is as much a necessity in the case of the eye as of any other organ for an easy and lasting performance of its function. No eyes, in my opinion, should be used more than an hour at the farthest in the act of reading or writing without an interruption of the gaze, and it would be better if several, if not many interruptions should take place in the same time.

This usually happens in the case of adults, for some reason or other; but children, in order to complete their tasks in an allotted time, are often compelled to use their eyes, without sufficient interruption, by the hour together. It would be impossible, and out of keeping with the condensed character of these remarks, to enumerate here all the diseases which may arise from prolonged tension of the eyes on near work; but there is one affection produced by it, which is so frequent in its occurrence and so unfortunate in its results, that I cannot refrain from quoting the remarks of the distinguished Prof. Donders. He says:

"The distribution of near-sightedness chiefly in the cultivated ranks points directly to its principal cause: tension of the eyes for near objects. Respecting this fact there can be no doubt.

"Three factors may here come under observation: 1. Pressure of the muscles on the eye-ball in strong convergence of the visual axes; 2. Increased pressure of the fluids, resulting from accumulation of blood in the eye in stooping; 3. Congestive processes in the eye which, tending to softening, give rise to extension of the membranes. Now, in connection with the causes mentioned, the injurious effect of fine work is, by imperfect illumination, still more increased.

"To this it is to be ascribed that in schools where, by bad light, the pupils read bad print or write with pale ink, the foundation of near-sightedness is mainly laid, which, in fact, is usually developed in these years."

But little will be said in reference to desks and seats, though it would seem they deserve the most careful consideration on the part of those in charge of the education of children, since a large portion of a child's life is spent behind the one and upon the other. It has been contended that every pupil should have a desk to suit his size, but this in many instances is impracticable for several reasons, but mainly on account of the expense. The chief idea to be borne in mind in the arrangement of desks is that they should be so constructed that the children can sit without becoming too rapidly wearied, and that they (the desks) should not be so low as to cause the body to bend forward, nor so high as to make studying difficult, as in writing for instance.

But of what use are all these proper arrangements at school, if as soon as the children get to their own homes, they write and study at tables that are inconvenient even for the grown members of the family? Of what benefit is the most superb illumination in the school-room, if the children, when at home, work several hours of the day in the corner of a badly-lighted chamber, and at night by the light of a flickering candle or lamp, used perhaps by five or six others of the family, and which is insufficient to properly light up the book or map of even one or two of the children? If we wish to protect the eyes of children during the time in which they are acquiring their education, give them above all things else, plenty of well-regulated light.

They should be prohibited using their books during twilight, by the light of the moon, and certainly should they be forbidden such dangerous use of their eyes as reading in bed.

Says a well-known writer: "It seems to me that the very etymology of the word education enforces the idea that the child is to grow better and stronger up through his school life; that by proper regulation of his diet and management at home; by properly lighted school-rooms and properly constructed desks, and a better regulation of his hours of study, he should represent a much higher type of life when he has reached the age of twenty-five, than when he is just taken in hand with the view of giving him book knowledge. We certainly should not damage the eye in the process of education, and I believe that the damage done to the eye is to be taken as an index of that which is done to the other organs of the body."

In conclusion, when every school house in the land, and every school-room and school desk shall have been properly constructed according to the most scientific investigations, and plenty of good light thrown upon books properly and plainly printed with good ink—when the habits of study of all children shall have been regulated, both in the school-room and at home, then do I feel convinced that while we may not be able to banish these particular eye diseases from the world, without doubt will we be able to reduce them in number and in degree of severity.

